# Microwave-Based Microfluidic Sensor for Non-Destructive and Quantitative Glucose Monitoring in Aqueous Solution

**DOI:** 10.3390/s16101733

**Published:** 2016-10-19

**Authors:** Thomas Chretiennot, David Dubuc, Katia Grenier

**Affiliations:** LAAS-CNRS, Université de Toulouse, CNRS, Toulouse 31031, France; thomas.chretiennot@gmail.com (T.C.); grenier@laas.fr (K.G.)

**Keywords:** microwave, microfluidic, sensor, glucose, glycaemia

## Abstract

This paper presents a reliable microwave and microfluidic miniature sensor dedicated to the measurement of glucose concentration in aqueous solution. The device; which is integrated with microtechnologies; is made of a bandstop filter implemented in a thin film microstrip technology combined with a fluidic microchannel. Glucose aqueous solutions have been characterized for concentration ranging from 80 g/L down to 0.3 g/L and are identified with the normalized insertion loss at optimal frequency. The sensitivity of the sensor has consequently been estimated at 7.6 × 10^−3^ dB/(g/L); together with the experimental uncertainty; the resolution of the sensor comes to 0.4 g/L. These results demonstrate the potentialities of such a sensor for the quantitative analysis of glucose in aqueous solution.

## 1. Introduction

In its recent edition of the Diabetes Atlas, the International Diabetes Federation estimated that the number of adults living with diabetes has reached to 366 million, representing 8.3% of the global adult population [1]. This number is projected to increase to 552 million people by 2030, or 9.9% of adults, which equates to approximately three more people with diabetes every 10 s. These figures explain why diabetes is predicted to become the seventh leading cause of death in the world by the year 2030 [2]. Nevertheless, a large-scale study [3] has proved that, with a constant blood glucose monitoring, patient can avoid any complication. The precise and regular knowledge of the blood-glucose level is consequently mandatory and, in conjunction with appropriate treatments, levels must be maintained in the range of 0.5 to 2 g/L. Currently, main glucose-monitoring techniques, which are based on electrochemical reaction, have demonstrated high accuracy and strong reliability [4]. However, these solutions lead to the destruction of blood samples.

Major efforts are consequently spent on developing techniques to measure the glucose concentration in blood non-invasively. Different electromagnetic liquid sensors have been developed for fluid characterization [5,6,7,8], for bio-liquids analysis [9,10,11], and more specifically for glucose monitoring applications [12,13,14,15] and demonstrated that microwaves are appropriate for aqueous solution analysis. These sensors are either based on cumbersome resonant structures, more sensitive to glucose variation or either miniature, for on-chip system integration [14,15] but requiring further resolution (both sensitivity and repetitiveness) improvement.

This paper presents the experimental demonstration of a miniature microwave sensor, which may be envisioned for glucose monitoring in aqueous solution without compromise on its sensitivity and resolution, and its application as a potential non-invasive blood glucose analysis method.

## 2. Sensor Description 

The biosensor is made of a quarter-wave length stub implemented in a thin film microstrip technology. The stub is connected to a microstrip feedline at one end and to the first electrode of an inter-digitated capacitor (IDC) at the other end. The second electrode of the IDC is grounded with a via. Such a structure behaves as a stopband filter and can conveniently be characterized in transmission. 

The device is implemented in a thin film microstrip (TFMS) technology constituted of a 20 µm thick SU8 layer. The photoresist SU8 layer, which acts as the TFMS substrate, is patterned to allow via connection of the IDC. Both strip line (on top of the SU8 layer) and ground plane (on the bottom of the SU8 layer) metallization are realized in gold (0.3 µm thick). The feedline is 54 µm wide in order to match a 50-ohm characteristic impedance. All gaps in the IDC are 10 µm wide. The length and width of the capacitor are 150 µm and 130 µm respectively.

**Figure 1:**
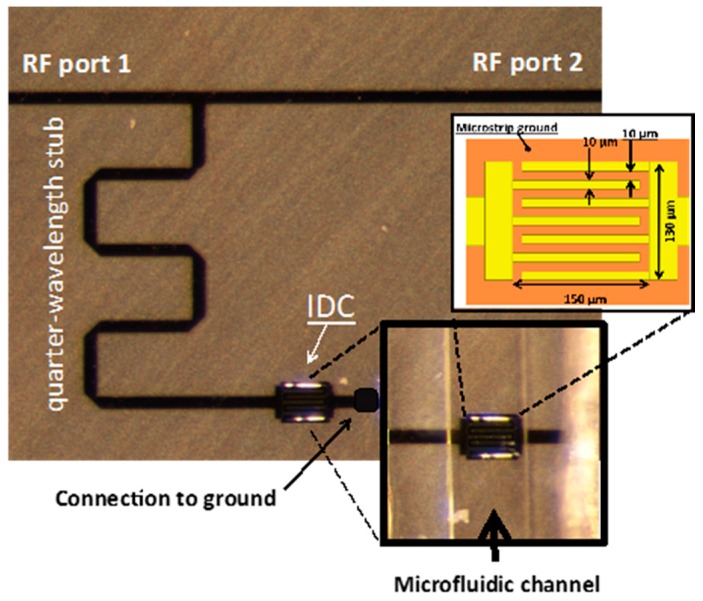
Fabricated biosensor consisting of a quarter wave-length stub implemented in a thin film microstrip technology. Inlets provide the detailed architecture of the inter-digitated capacitor (IDC) equipped with the microfluidic channel and the IDC dimensions.

A microfluidic channel made of polydimethylsiloxane (see [16,17] for further details) is placed on the IDC, as it corresponds to the area where the electric field is of highest intensity at the resonant frequency and it enables the most efficient electric field/fluid coupling [7]. Such microfluidic channel is in charge of the fluid conveying over the sensing area and presents the great advantage to be compatible with microfluidics capabilities for lab-on-a-chip [18]. The cross-section of the microfluidic channel is 50 µm per 50 µm. Figure 1 shows a top view of the device fabricated in the clean room at LAAS-CNRS. Inserts in Figure 1 provide the detailed architecture of the interdigitated capacitor equipped with the microfluidic channel and the IDC dimensions.

**Figure 2:**
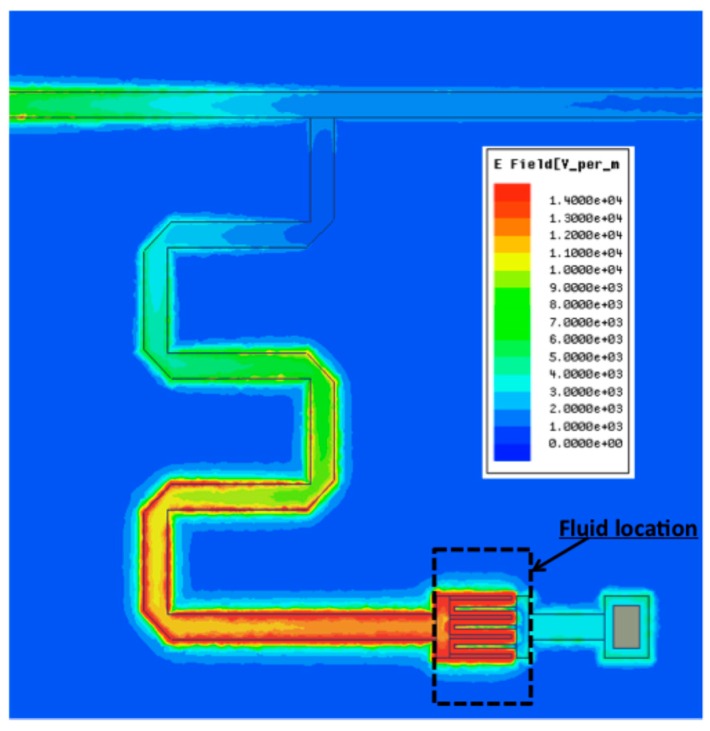
Simulated electric field intensity at the resonance.

**Figure 3:**
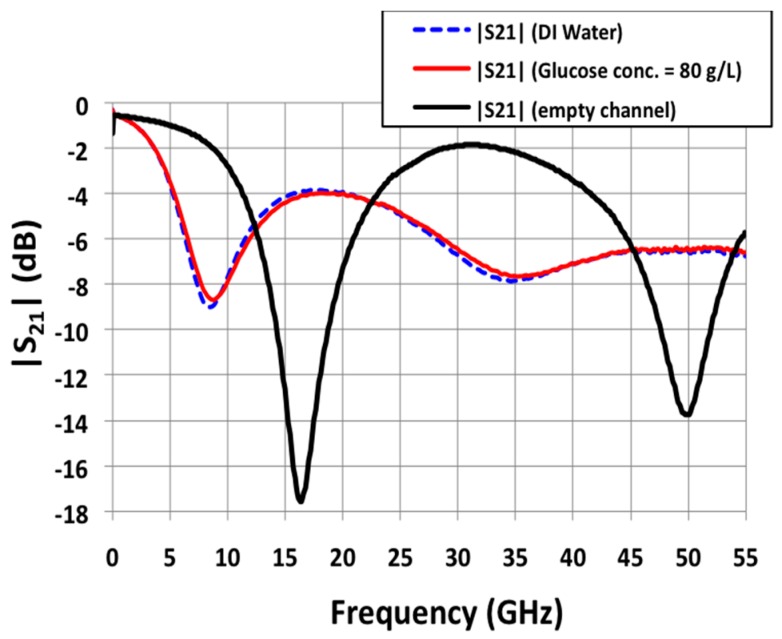
Measured S21 spectra in the frequency range [0; 55] GHz for deionized water (reference liquid in this study) and a glucose solution at 80 g/L.

Figure 2 shows a 3D full wave simulation of the stub at the resonant frequency of 7.5 GHz (see Figure 3 in Section 3). Simulations have been realized with Ansys HFSS^®^. The color code confirms that the electric field of highest intensity is concentrated in the IDC. 

## 3. Experimental Results

**Figure 4:**
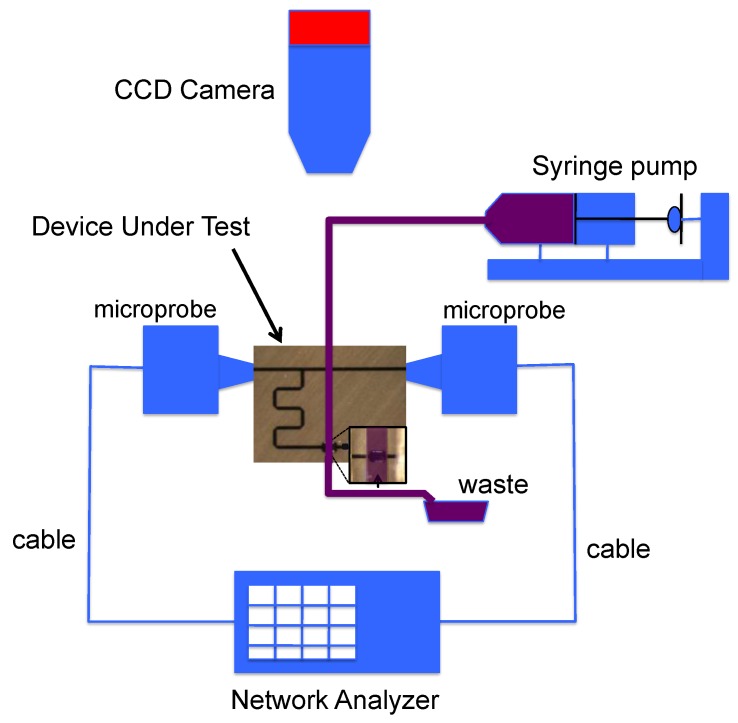
Block diagram of the RadioFrequency (RF) measurement setup.

Measurements are made on wafer directly with RadioFrequency (RF) microprobes. The block diagram of the RF measurement setup is presented in Figure 4. Measurements are preceded by a SOLT calibration process in the [0; 55] GHz frequency band which sets the reference planes at the tips of the microprobes. Fluid samples are then injected one-by-one thanks to a syringe pump, characterized when the liquid is stabilized over the sensing zone and evacuated. During the measurement, we check with a microscope that any bubble remains in the sensing area. Temperature is also kept constant at 20 °C thanks to a thermally controlled chuck in order to avoid any shift in the dielectric properties of the glucose solutions due to significant temperature variations.

Figure 3 shows the measured magnitude in dB of the S21 parameters versus frequency for different channel filling. Such parameter corresponds to the transmission of the stopband filter, which is expected to be (highly) sensible to the permittivity characteristics of the liquid loaded in the microfluidic channel. When the channel is empty (black curve in Figure 3), the biosensor is characterized by a resonant frequency of 16.4 GHz, associated rejection of −17.6 dB and quality factor of 1.2. Full-wave simulations gave a resonant frequency of 16.8 GHz, associated rejection of −17.7 dB and quality factors of 1.1. Such a good agreement between simulations and measurements confirms the good operation of the device.

A series of six aqueous glucose mixtures has been measured on our biosensor. The glucose concentration varied from 80 g/L (high concentration) to 5 g/L (medium concentration, five time higher than physiological concentrations). Figure 3 also shows the measurements corresponding to deionized water (dashed blue) and to the glucose mixture at 80 g/L (red). This figure clearly demonstrated, based on the glucose dependent permittivity of the injected aqueous solution, a slight but significant contrast (shift in resonant frequency and associated rejection) between pure DI water and 80 g/L glucose aqueous solution.

**Figure 5:**
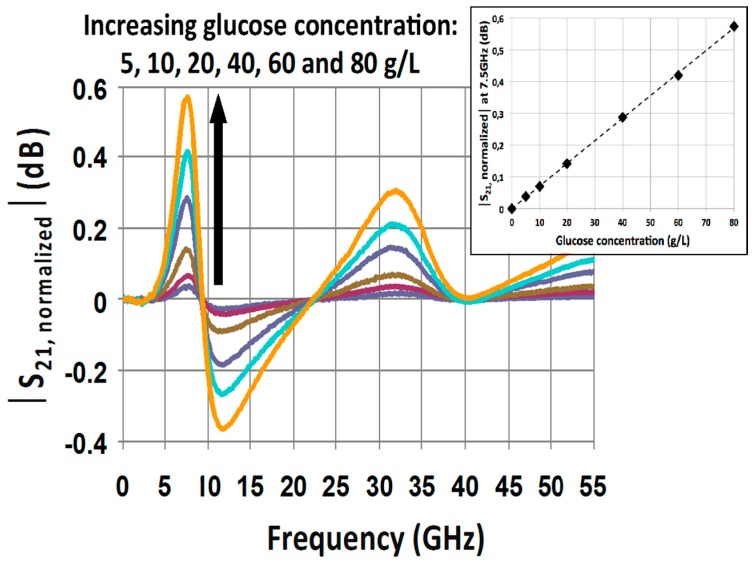
Measured normalized spectra S21 for the highest eight concentrated glucose solutions in the same frequency range.

In order to exacerbate the influence of glucose concentration on the sensor response, each S21 signature associated to a glucose mixture is normalized by the S21 parameter of the reference liquid (deionized water in this study). Practically speaking, that means that a sample of water must be measured directly before each sample of glucose mixture. Figure 5 shows the resulting normalized |S21,normalized| parameters associated to each glucose mixture. This figure clearly facilitates the distinction of each glucose concentration and points out that at 7.5 GHz, the contrasts between the different glucose concentrations are maximized. The insert in Figure 5 presents the values of |S21,normalized| parameter at 7.5 GHz as a function of the glucose concentration.

This result reveals a linear relationship between the selected microwave readout |S21,normalized| parameter at 7.5 GHz and the glucose sensor for glucose concentration up to 80 g/L. The high linearity of the sensor’s response (the coefficient of correlation is higher than 0.99) validates that microwave sensing of biomolecules in aqueous solution represents a reliable and predictable technique. 

**Figure 6:**
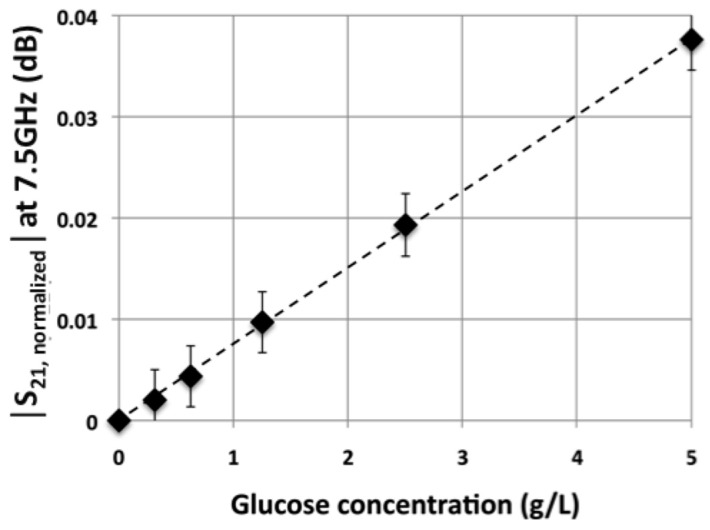
Modulus of the normalized S21 parameter at 7.5 GHz as a function of the glucose concentration.

In order to demonstrate the ability of such a technique to sense glucose in aqueous solution for blood hyperglycemia applications, further investigations have been carried out considering glucose concentration at a physiological concentration around 1 g/L. Figure 6 presents the values of |S21,normalized| parameter at 7.5 GHz as a function of the glucose concentration for values ranging from 0.31 g/L to 5 g/L. Various concentrations have been obtained by successive dilution by a factor two of a mother solution and measurements have been repeated 10 times.

Once again, the Figure 6 demonstrates a linear response of the sensor for the considered range of glucose’s concentrations and down to physiological glucose concentration in blood. 

## 4. Discussion on the Chieved Resolution

From Figure 6, the sensitivity S of the sensor can be defined and evaluated as follows:

Δ|S21,normalized|(7.5 GHz)/Δ[glucose] = 7.6 × 10^−3^ dB/(g/L) (1)

Moreover, repetitive tests enabled the assessment of the experimental uncertainty: δ = 3 × 10^−3^ dB. Sensitivity and experimental uncertainty allow the definition of the biosensor resolution (R), that is to say the smallest variation of glucose concentration that the sensor is able to quantify:

R = δ/S = 3 × 10^−3^/7.6 × 10^−3^ = 0.4 g/L (2)

Such a resolution indicates that this thin film miscrostrip biosensor has reached the same performances as dielectric resonant-based devices [12,13,15].

**Figure 7:**
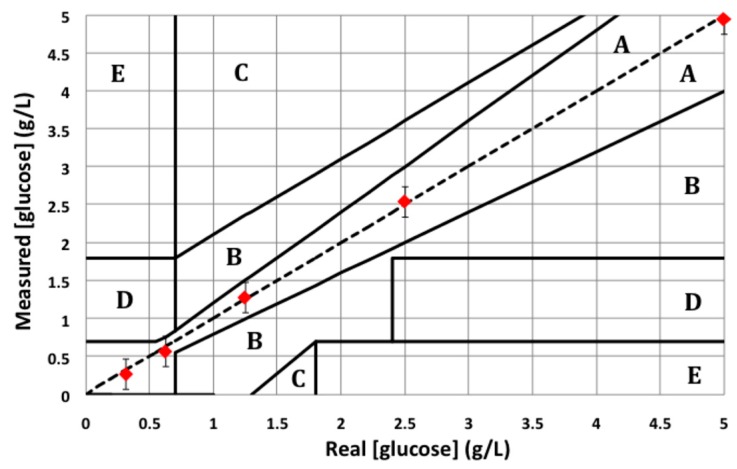
Clarke error grid established with the lowest five glucose solutions measured on the presented biosensor.

In order to further evaluate the sensor reliability for glucose monitoring applications, measured glucose concentrations are plotted using the Clarke error grid. The Clarke error grid has been worked out in 1987 by the so-called biologist Clarke in order to evaluate the reliability of the commercial glucometers [19]. The Clarke error grid is given in Figure 7. X-axis provides real glucose concentrations; Y-axis provides the measured glucose concentrations. Clarke divided the grid into five different areas named A, B, C, D and E. Area A is the optimal area where the error on the glucose concentration does not exceed 20%, which is considered as acceptable for the screening of human glycaemia. As far as area B is concerned, error on glucose concentrations exceeds 20% but is not detrimental for patients. Areas C, D and E are the hazardous ones: such an error on the glycaemia measurement may endanger the patient's health.

Figure 7 shows the lowest five glucose solutions that have been measured on our glucose biosensor in the Clarke error grid. This figure demonstrates the potentialities of our sensor for the determination of the human glycaemia as all the measured concentrations lie down in the area A.

Moreover, since the electromagnetic (EM) field can penetrate different tissue materials at microwave frequencies, especially the different layers of human skin, and combined to the non-destructive ability of the technique, the demonstrated results together with others [20] contribute to establish the potential of the microwave technique for non-invasive blood glucose monitoring.

## 5. Conclusions

This paper has experimentally demonstrated the reliable operation of a microwave and microfluidic based sensor dedicated for glucose concentration quantification (down to 0.4 g/L) in aqueous solutions. Combined with its non-destructive ability, the microwave sensing is consequently identified as an attractive technique for blood parameters monitoring.
